# Understanding Contemporary Determinants of Antimicrobial Resistance: Towards One Health Literacy and Differentiated Responsibility

**DOI:** 10.3390/antibiotics15100988

**Published:** 2026-10-06

**Authors:** Gordana Belamarić, Svetlana Mladenović Janković, Rada Sandić Spaho, Gordana Marković

**Affiliations:** 1 Institute of Public Health of Belgrade, 11000 Belgrade, Serbia; gordana.belamaric@zdravlje.org.rs (G.B.); svetlana.jankovic@zdravlje.org.rs (S.M.J.); 2 Faculty of Nursing and Health Science, Nord University, 8049 Bodø, Norway; 3 Faculty ECM, Alma Mater Europaea University, 2000 Maribor, Slovenia; gordana.markovic@almamater.si

**Keywords:** antimicrobial resistance, One Health, AMR literacy, determinants, antimicrobial stewardship

## Abstract

Antimicrobial resistance (AMR) is commonly framed as a consequence of antimicrobial use, yet many conditions that shape resistance arise before treatment is considered and outside clinical care. This review examines contemporary determinants of AMR across human health, animal health, food production, environmental systems, and wider social and institutional contexts. Determinants are organized around common pathways, such as infection burden, antimicrobial exposure, selection pressure, transmission, and systems’ capacity to prevent and control resistance. This pathway-based perspective helps identify critical intervention points in diagnostics, infection prevention, vaccination, water and sanitation, biosecurity, wastewater management, governance, and emergency preparedness. It also shows why surveillance conducted within separate sectors may miss interactions at human, animal, and environmental interfaces. The synthesis supports a broader understanding of AMR literacy that extends beyond knowledge and individual antibiotic behavior to include recognition of interacting determinants, opportunities for prevention, and the different capacities of actors to influence them. Shared responsibility should therefore be understood as differentiated responsibility among individuals, professionals, institutions, industry, and governments. By linking determinants with the pathways through which they act, potential points for intervention, and different levels of responsibility, this review provides a framework for more coherent One Health surveillance, prevention, preparedness, and policy responses.

## 1. Introduction

In 2021, bacterial AMR was associated with an estimated 4.71 million deaths globally, including 1.14 million deaths directly attributable to resistance, underscoring the scale of its current health burden [1]. The increasing prevalence and severity of antimicrobial resistance (AMR) worldwide, including resistance affecting all major classes of clinically approved antibiotics, have raised serious concern that effective treatment options may progressively diminish and that medicine may be moving towards a “post-antibiotic era” in which infections may once again become difficult or impossible to manage effectively [2,3]. Clinically significant resistance often emerges within a decade of introducing a new antibiotic, limiting its long-term effectiveness and requiring cautious use [4]. Innovations are costly, and developing a new antibiotic typically takes more than a decade, underscoring the need for sustained investment to increase the future availability of effective therapies for infections caused by multidrug-resistant bacteria. Although the World Health Organization has identified antimicrobial resistance as one of the most important public health threats of the twenty-first century [5], current global action still appears insufficient to address the problem’s urgency. Without coordinated and global action, antimicrobial resistance could have severe medical, social, and economic consequences worldwide [6,7]. Research shows that microorganisms have produced antibiotics for millennia, suggesting that resistance should be understood as an evolutionary phenomenon rather than just as a consequence of antimicrobial use [8]. Spagnolo et al. see examining the ecological and evolutionary circumstances in which microorganisms naturally produce antibiotics as a key to gaining further insight into the development of resistance and its control over time [4]. The practical question is therefore broader than how to reduce antimicrobial use, but how health, food, animal, environmental, and social systems create or interrupt the pathways through which AMR emerges and spreads. Although biological and evolutionary mechanisms such as horizontal gene transfer, mobile genetic elements, biofilm formation, co-selection, and sub-inhibitory antimicrobial exposure are central to the emergence and persistence of resistance, a detailed review of these mechanisms is beyond the scope of this article. The present review focuses primarily on behavioral, clinical, social, institutional, animal, and environmental determinants that shape antimicrobial exposure, transmission, and opportunities for prevention within a One Health framework.

Relevant literature for this narrative review was identified through searches of PubMed and Google Scholar, supplemented by reference list screening and reports or policy documents from major international organizations, including WHO, WOAH, FAO, UNEP, and ECDC. Searches focused primarily on literature published during the past decade, while earlier landmark publications were included where relevant to the historical or conceptual explanations. Search concepts included antimicrobial resistance combined with terms related to antimicrobial use and stewardship, human and animal health, food production, wastewater, climate change, health emergencies, social and institutional determinants, One Health, and AMR literacy. English-language peer-reviewed articles, reviews, relevant observational studies, and authoritative international reports were considered when they contributed directly to the determinants and pathways discussed in this review. Publications with limited relevance to the scope of the review were excluded. The literature search was updated throughout manuscript preparation, with the final search conducted on 28 August 2026. While antimicrobial resistance encompasses bacterial, fungal, viral, and parasitic resistance, the present review primarily addresses antibacterial resistance and the determinants associated with antibiotic use, selection, and transmission.

## 2. From Antimicrobial Stewardship to One Health Literacy

The concept of antimicrobial stewardship was introduced as a way to protect the long-term effectiveness of antimicrobials by improving decisions about their use and avoiding unnecessary treatment [8,9]. This early view of antimicrobials as a valuable therapeutic resource has since evolved into a broader concept of collective responsibility, aimed at balancing appropriate access and rational use while preserving antimicrobial effectiveness for the future across human and animal health systems, food production, and the environment [9,10]. Evidence-based policies and practices that ensure responsible use of antimicrobials are important for reducing unnecessary exposure and limiting the spread of resistance [11,12].

AMR literacy has commonly been approached through awareness, knowledge, attitudes, and behaviors related to antibiotics and antimicrobial resistance, with considerable attention to appropriate antimicrobial use and educational interventions [13,14]. More recent developments in One Health literacy offer a broader perspective, including the understanding that antimicrobial resistance is shaped by social, economic, institutional, and national contexts, not only by individual decisions made by patients or prescribers [14,15]. Antimicrobial stewardship has been framed as a collective responsibility involving actors at different levels and in different contexts [10]. From this perspective, shared responsibility for AMR should be understood as differentiated rather than uniform responsibility: individuals, health professionals, institutions, industry, and governments all have roles, but their capacity to influence specific determinants and the actions available to them differ. The greatest opportunities for AMR prevention often lie in infection prevention, vaccination, diagnostics, sanitation, biosecurity, environmental control, and strengthening the overall resilience of health systems. This review therefore examines selected contemporary determinants that influence the emergence, transmission, and control of AMR across human, animal, environmental, and social systems. It considers how understanding their interactions may contribute to a broader conception of One Health literacy and shared responsibility. For the purposes of this review, One Health-oriented AMR literacy refers to the capacity to understand AMR as a result of interconnected human, animal, environmental, and social processes, to recognize major pathways through which resistance emerges and spreads, and to identify opportunities for prevention and the roles of different actors. Institutional capacity, governance, and infrastructure are not considered components of literacy itself, but conditions that may enable or constrain the translation of knowledge into effective action.

## 3. Human and Health System Determinants of AMR

The development and spread of AMR are shaped by a wide range of interconnected determinants including behavioral, clinical, organizational, social and environmental conditions [16,17,18]. Understanding these determinants and their interconnections is essential for moving from a narrow focus on antimicrobial use to a broader One Health interpretation of the processes that sustain resistance. In the human population, relevant factors include individual behavior, prescribing practices, access to medicines and diagnostics, health system organization, and broader social conditions. Non-prescription antibiotic use remains a major contributor to inappropriate antimicrobial use and spread of AMR [19]. Despite regulations restricting over-the-counter access in many countries, studies consistently report that a proportion of the population continues to obtain and use antibiotics without prescription, either through pharmacies or their informal sources [19,20,21,22]. These findings suggest that legal restrictions are unlikely to be sufficient without effective enforcement, accessible medical care, and informed public decision-making.

Excessive antibiotic use in human medicine is closely related to inappropriate prescribing, particularly in primary health care, where prescribing decisions are shaped by interactions between physicians and patients as well as by wider organizational and sociocultural factors [23,24]. Patient expectations and requests for antibiotics may contribute to unnecessary prescribing, while physicians may face limited consultation time, insufficient support for patient education, and difficulties in challenging established expectations, creating a cycle that sustains antibiotic use even for viral infections [25]. Mutual trust between patients and health professionals is essential for effective communication and AMR stewardship. Still, its role is complex, as greater trust may facilitate acceptance of appropriate information while also increasing patients’ expectations that antibiotics will be prescribed [26,27,28].

Another important challenge is limited access to timely and accurate microbiological diagnostics, which can lead to greater reliance on empirical treatment with broad-spectrum antibiotics. Diagnostic stewardship therefore plays an important role in AMR control, as it enables selection of the appropriate drug and earlier adjustment to more targeted antimicrobial treatment [29]. Substantial differences in antibiotic consumption between European countries, along with the observed association between higher antimicrobial use and higher resistance levels, further indicate that prescribing practices are strongly influenced by national health systems, professional norms, and local patterns of antimicrobial use [30]. Effective stewardship therefore requires context-specific interventions that combine continuous education and support for prescribers with improved public understanding, while strengthening communication and shared decision-making between patients and health professionals [31,32].

These interactions also illustrate why understanding AMR requires more than factual knowledge about antibiotics and must include the clinical, organizational, and social conditions in which decisions about their use are made. Inadequate infection prevention and control, especially in health care settings such as hospitals, intensive care units, and long-term care facilities, can enable the rapid spread of resistant microorganisms, especially when hand hygiene, isolation, screening, and other infection prevention and control measures are not fully and consistently implemented [33]. Infection prevention is therefore central to the response to AMR in human medicine. Poor access to safe water, sanitation, and hygiene can increase infection incidence and transmission, including infections caused by resistant microorganisms. While the resulting higher infection burden may increase antimicrobial use, improvements in water, sanitation, and hygiene are important components of efforts to reduce the emergence and spread of AMR [34,35]. Together, these findings support antimicrobial use, diagnostic uncertainty, and infection transmission as comparatively well-established and modifiable determinants of AMR.

## 4. AMR Stewardship During Health Emergencies

The COVID-19 pandemic made the interdependence of human, animal, and environmental health more visible. It prompted renewed attention to One Health literacy, governance, and system leadership as components of preparedness and response [14,36]. The COVID-19 pandemic demonstrated the complexity of antimicrobial stewardship during public health emergencies, when diagnostic uncertainty, pressure on health systems, and concerns about secondary infections contributed to extensive antibiotic use, with potential consequences for AMR [36]. In critically ill patients, broad-spectrum antibiotics were frequently initiated empirically while awaiting diagnostic results, reflecting the need for immediate treatment under conditions of clinical uncertainty [37,38,39]. Crisis conditions may also influence antimicrobial use through psychological distress, fear, uncertainty, and altered perceptions of health risks, which can promote self-medication and preventive antibiotic use, particularly when knowledge is limited or access to professional guidance is disrupted [40,41]. The pandemic therefore clearly demonstrated that AMR is also influenced by health system resilience, access to care, and the capacity to maintain stewardship under exceptional conditions. Future emergency preparedness should integrate antimicrobial stewardship, surveillance, infection prevention, and risk communication into coordinated One Health planning to minimize avoidable antimicrobial use during crises. Beyond pandemics, other emergencies can also create conditions in which AMR risk may intensify. A systematic review of studies conducted in conflict settings found high rates of infection and colonization with multidrug-resistant bacteria, indicating that war and conflict can also create conditions that facilitate the persistence and dissemination of resistant pathogens [42].

Conflict may simultaneously disrupt health infrastructure, infection prevention and control, water and sanitation services, microbiological diagnostics, and antimicrobial supply chains, while displacement can increase opportunities for infection transmission [12,43]. Evidence from pandemic and conflict settings suggests that antimicrobial stewardship during emergencies depends strongly on health system organization and resilience, and that it should be incorporated into preparedness and response planning.

## 5. Animal Health, Food Production, and AMR

Available evidence indicates that animals and animal-derived products can contribute to the human burden of resistant infections. Still, the magnitude of this contribution remains uncertain, and the extent of variation across species, exposure routes, and pathogens varies [44]. Although evidence on the direct contribution of animal sources to human AMR is still limited, the number of studies addressing these pathways is growing [44]. Further research is therefore needed to clarify the relative contribution of different animal sources within the broader One Health transmission network. Antimicrobial use and animal production conditions, including hygiene, biosecurity, and infection prevention, influence the contribution of food-producing animals to AMR by shaping disease burden, the need for antimicrobial treatment, and the presence of resistant bacteria in animal waste [45]. Antimicrobial resistance in food-producing animals has become an increasingly important component of the global AMR burden, particularly in low- and middle-income countries where livestock production is expanding rapidly. Van Boeckel et al. showed that resistance in animal bacterial pathogens increased substantially between 2000 and 2018, with major hotspots identified in China and India and emerging areas of concern in countries such as Brazil and Kenya [46]. These findings indicate that AMR in animal populations follows distinct geographic and production-related patterns and may intensify alongside the growth and industrialization of animal production [46]. Vaccination, biosecurity, appropriate hygiene, good husbandry practices, adequate nutrition, and access to diagnostics can reduce the disease burden and, consequently, decrease the need for antimicrobial treatment [47]. Resistant microorganisms and resistance determinants originating in animal populations may reach humans through direct contact, the food chain, and environmental pathways, including contamination associated with animal production and waste [48]. Although interventions aimed at reducing antibiotic use in food-producing animals appear to have a measurable effect on antimicrobial resistance, the goal is to minimize unnecessary exposure while preserving access to effective treatment when required for animal health and welfare. Current recommendations emphasize reducing the use of medically important antimicrobials and avoiding their routine use for growth promotion or disease prevention in healthy animals [47,48,49]. World Organisation for Animal Health (WOAH) strongly advocates limiting antimicrobial use in animals to legitimate veterinary purposes. Although the use of antimicrobials for growth promotion has declined, it is still reported in nearly one-quarter of WOAH member countries [47].

A systematic review and meta-analysis found that restrictions on antibiotic use were associated with lower levels of antibiotic-resistant bacteria in these animals. At the same time, a smaller body of evidence suggested similar benefits in humans with direct occupational or other close exposure to food-producing animals [50]. Evidence for effects in the general human population was less conclusive, largely because relatively few studies had examined this pathway. Further longitudinal, cohort, and case–control studies are needed to clarify the risk and direction of transmission. Still, the animal dimension of AMR should not be limited to livestock, since evidence also indicates exchange of resistant bacteria between humans and companion animals, with transmission potentially occurring in both directions [50]. The close and repeated contact between humans and companion animals therefore represents an important interface for studies of AMR transmission.

Veterinary antimicrobial stewardship should be considered together with measures that reduce the need for antimicrobial treatment in the first place. Improved diagnostics can support more targeted therapy, while vaccination, biosecurity, hygiene, appropriate husbandry, and disease prevention can reduce infection burden and subsequent antimicrobial demand in animal populations. These measures are particularly relevant in food-producing animals, where antimicrobial use, animal health, production practices, and environmental release are closely connected [45,47,50]. Companion animals represent an additional human–animal interface, with evidence suggesting that resistant bacteria may be exchanged in both directions, although the magnitude and direction of transmission remain incompletely understood [51]. Food-chain and environmental pathways, including animal waste and aquaculture systems, further illustrate how animal-health determinants may extend beyond the point of antimicrobial use and contribute to broader One Health AMR dynamics.

## 6. Agricultural and Environmental Determinants of AMR

Global responses to AMR have been traditionally concentrated on human health, but current strategies increasingly recognize the need for coordinated action across human, animal, plant, and environmental sectors [51]. Although the environment plays several interconnected roles in the evolution and dissemination of AMR, important environmental pathways remain insufficiently characterized [52]. Environmental systems can create conditions that favor the selection and evolution of resistance, serve as reservoirs for resistant bacteria, facilitating their movement between environmental, animal, and human compartments [53]. The contribution of agricultural soils and diverse animal populations to the emergence and spread of AMR remains incompletely understood, with important specific evidence gaps across livestock, equine, and companion animal systems, highlighting the need for more comprehensive and more differentiated surveillance across these interconnected One Health domains [53,54,55]. Therefore, a One Health approach enables more coherent surveillance, prevention measures, antimicrobial stewardship, and response. Although plant health is also part of the broader One Health context of AMR, this review does not examine antimicrobial use and resistance in plant pathogens in detail, as it focuses primarily on determinants related to human and animal health, food production, and environmental pathways.

Martak et al. suggest that high-income countries, stronger sanitation systems, and tighter control of human and industrial waste appear to limit the transmission of resistant bacteria between environmental, animal, and human reservoirs, with person-to-person spread remaining the dominant route [56]. In low- and middle-income countries, weaker sanitation infrastructure, high population density, and close contact with animal production systems may create more opportunities for exchange between these reservoirs [46]. Berendonk et al. emphasize the importance of identifying critical control points within environmental pathways of AMR, where targeted interventions may reduce the release, persistence, or further dissemination of resistant bacteria. Such control should limit environmental contamination and interrupt transmission between environmental, animal, and human reservoirs [57]. Particularly relevant control points include wastewater treatment, manure and sewage sludge management, aquaculture effluents, and waste streams from pharmaceutical manufacturing. Interventions at these points are important for reducing the release of antimicrobial residues, resistant bacteria, and resistance genes before they enter wider environmental systems, representing a fundamentally preventive approach to AMR control [54,58].

Climate change has become an increasingly important environmental determinant of AMR, influencing both conditions that favor resistance and the capacity of health and public health systems to prevent and control its spread [59]. Climate change may contribute to the emergence and spread of AMR through several interconnected pathways, particularly during extreme weather that can damage health care and sanitation infrastructure and place additional pressure on already strained health systems [60]. Strengthening climate resilience within health and public health systems is therefore increasingly relevant to efforts to limit the development and transmission of antimicrobial resistance. Higher outdoor temperatures and extreme heat have been associated with increased resistance in several clinically important pathogens [61,62]. Associations between temperature, climate-related factors, and AMR should be interpreted cautiously, as ecological analyses may be affected by differences in antimicrobial use, population density, sanitation, health system capacity, surveillance practices, and other contextual factors that vary geographically.

From a One Health perspective, aquaculture provides a clear example of how climate-related conditions may interact with AMR across animal, environmental, and food production systems. Antimicrobial resistance levels in aquaculture have been associated with temperature and national climate vulnerability, while bacterial infections in farmed aquatic animals have been linked to higher mortality at warmer temperatures, representing new challenges for disease control and antimicrobial use [63]. More broadly, a systematic review identified multiple pathways connecting climate change and AMR across human, animal, and environmental systems, but also found that the evidence remains fragmented and that many of these relationships require stronger empirical confirmation [59]. Environmental- and climate-related influences are increasingly supported by evidence, although the magnitude and direction of their contribution remain difficult to quantify across settings and require further research.

## 7. Social and Institutional Determinants of AMR

It remains insufficiently understood how social determinants of health and environment further influence the emergence and spread of AMR [17]. These broader determinants operate at different levels, from individual social conditions to the quality and functioning of institutions and public systems. Governance is one example of potential wider influence. Across European settings, higher levels of corruption and poorer institutional quality have been associated with greater antibiotic consumption and higher levels of antimicrobial resistance in general, suggesting the importance of the capacity to regulate antimicrobial use, enforce standards, and maintain effective health and public systems [64,65,66]. Social inequalities may contribute to AMR through similarly complex pathways. Greater income inequality across European countries has been correlated with higher resistance in several major bacterial species, indicating that the distribution of socioeconomic resources may matter independently of overall national wealth [67]. More recently, a global analysis of 70 countries found an association between higher female educational attainment and lower antibiotic consumption, suggesting that inequalities may influence patterns of antimicrobial use [68]. At the community level, socioeconomic deprivation could be associated with a higher prevalence of resistant organisms [69]. Part of the evidence linking social, economic, and institutional conditions with AMR is observational and should therefore be interpreted as associative rather than causal. Cross-country and regional comparisons may be influenced by differences in antimicrobial consumption, health system organization, surveillance intensity, laboratory capacity, national income, and data completeness. Residual confounding is also likely, as many of these factors are interrelated and may influence both exposure to AMR determinants and the likelihood that resistance is detected and reported.

These findings suggest that a contemporary understanding of AMR requires attention to the social and institutional conditions that may influence exposure to infection, access to health care, antimicrobial use, regulatory implementation, and the capacity of health systems to prevent and control resistance. This places greater emphasis on prevention at the community level and on strengthening health care and public health systems globally, since their capacity to reduce infection risk, support appropriate antimicrobial use, and maintain effective surveillance and control can shape the conditions in which AMR develops and spreads. These factors are therefore better interpreted as broader contextual or amplifying determinants, with the current evidence remaining largely associative rather than causal.

## 8. National Experiences and Transferable Lessons

Experiences from different countries are particularly informative when considered together, as they show that AMR can be addressed at multiple points within the same broader system. Surveillance, regulation, professional practice, public communication, diagnostic support, and changes in animal production can all contribute, while sustained progress depends on their coordination over time.

The Swedish experience illustrates how sustained and comprehensive national action can translate into measurable progress in antimicrobial stewardship. Since the establishment of a national strategic framework for AMR in 1995, Sweden has gradually and consistently implemented coordinated measures across surveillance, prescribing, prevention, and professional practice, contributing to comparatively low antibiotic use and positioning the country as a leading example of successful AMR control [70]. This experience suggests that effective antimicrobial stewardship depends on long-term commitment and constant implementation of mutually reinforcing measures. In this respect, the Swedish experience may provide a useful roadmap for other countries seeking to adapt and implement similar principles of good practice within their own contexts.

Comparable experiences from other European countries show that progress can be achieved through different approaches. Danish Integrated Antimicrobial Resistance Monitoring and Research Programme was established in 1995, integrating surveillance of antimicrobial consumption and resistance across humans, animals, and food [71]. Evidence generated through this system supported restrictions on antimicrobial use in food animals and enabled the effects of these measures on resistance to be monitored over time.

In the Netherlands, a combination of regulatory and voluntary measures with explicit reduction targets was associated with a substantial decrease in antimicrobial use in farm animals over a five-year period, demonstrating the potential of coordinated action within the livestock sector [72].

France provides a different example, where a nationwide program combining public communication, professional education, and improved diagnostic support was associated with a significant reduction in community antibiotic prescribing over five years, with the largest decline among children [73]. Together, this evidence indicates that effective national responses may use different entry points. At the same time, their impact depends on sustained surveillance, regulation, professional practice, prevention, and public engagement that reinforce one another over time.

Examples from other health system contexts further illustrate the importance of adapting AMR strategies to national capacities and priorities. Thailand has implemented a multisectoral national AMR strategy that combines surveillance, regulation of antimicrobial distribution, stewardship, public awareness, and action across human, animal, food, and environmental sectors. Antimicrobial consumption in animals declined substantially during implementation of the strategy, although progress across other targets was more uneven [74]. Transferable principles of AMR control need to be adapted to the resources, governance structures, and surveillance capacity of each setting.

## 9. From AMR Determinants to Prevention and Shared Responsibility

**Figure 1:**
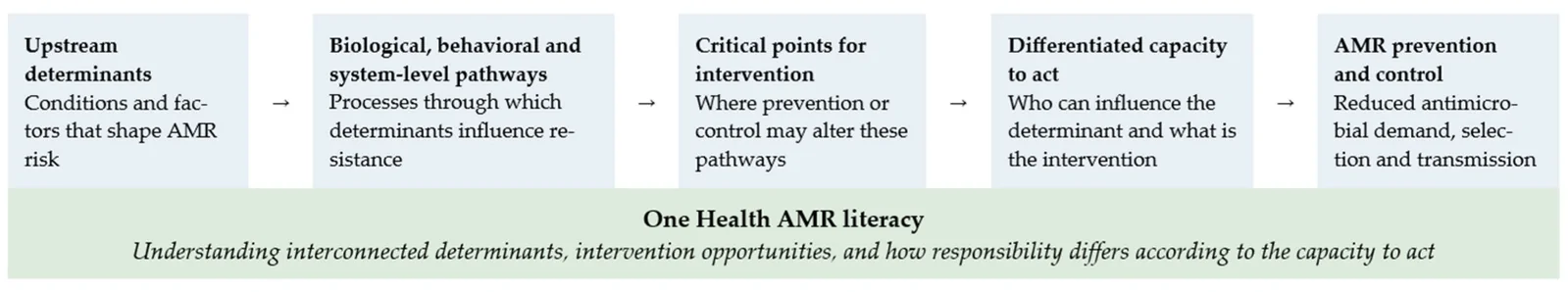
From AMR determinants to action: linking One Health literacy and differentiated responsibility.

Evidence across human health, animal production, environmental systems, and broader social and institutional contexts shows that AMR is influenced by interacting determinants that affect infection burden, antimicrobial exposure, transmission, and the capacity to prevent or control resistance. Contemporary AMR literacy should therefore incorporate an understanding of these relationships, the points at which preventive action is possible, and the different levels of responsibility involved. From this perspective, a One Health approach broadens AMR literacy beyond individual antibiotic-related behavior towards a better understanding of how upstream determinants act through shared pathways, where critical intervention points lie, and how capacity to act differs across sectors (Figure 1).

Determinants could be understood as upstream conditions or factors that shape the risk of AMR, including, for example, inadequate sanitation, limited access to diagnostics, inappropriate prescribing, and socioeconomic disadvantages. Pathways, in contrast, refer to the biological, behavioral, or system-level processes through which these determinants can influence the emergence and spread of resistance. It is important to notice that different determinants may converge on the same pathways, particularly increased infection burden, antimicrobial exposure, selection pressure, transmission, and reduced capacity for prevention and control. Table 1 summarizes selected examples of AMR determinants discussed in this review, together with the main pathways through which they may act and corresponding critical points for intervention.

Differentiated responsibility reflects the fact that actors differ in both their capacity to influence AMR determinants and the types of interventions available to them. Individuals and health professionals can influence antimicrobial use and preventive behavior, whereas determinants such as sanitation infrastructure, pharmaceutical emissions, agricultural regulation, surveillance capacity, and emergency preparedness largely depend on institutional, industry, or governmental action. Identifying the actors able to intervene at specific critical points therefore helps translate shared responsibility into more concrete and actionable roles.

The reviewed evidence is not equally strong across all determinants. The role of antimicrobial exposure, inappropriate prescribing, infection burden, and transmission within health care and animal production systems is comparatively well established, and several corresponding interventions, including antimicrobial stewardship, infection prevention, vaccination, improved diagnostics, and biosecurity, are supported by empirical evidence. Other factors, such as socioeconomic disadvantages, governance, climate-related pressures, and some environmental pathways, appear to act mainly as broader or amplifying determinants, but much of the available evidence remains observational and context-dependent. Several cross-sectoral pathways, including those involving environmental reservoirs, companion animals, and complex transmission between human, animal, and environmental systems, remain uncertain in terms of their relative contribution to AMR. These differences are important because some determinants are directly modifiable through targeted interventions, whereas others are more structural and require sustained institutional, regulatory, or multisectoral action.

## 10. Limitations of the Review

The interpretation of this review should consider several limitations. Evidence is unevenly distributed across One Health domains, with substantially stronger and more consistent data for human health and antimicrobial use than for several environmental, animal, social, and institutional determinants. The country examples were selected to illustrate practices that have generated useful lessons for AMR prevention and control rather than to support direct cross-country comparison. Differences in health-system organization, governance, regulatory frameworks, surveillance capacity, resources, and local patterns of antimicrobial use mean that successful measures cannot be transferred between settings and require adaptation to national and local contexts. For a few broader determinants, particularly those related to governance, socioeconomic conditions, climate, and environmental exposure, the evidence is largely observational and does not allow firm causal inference. In addition, the narrative design of this review involved a selective synthesis of literature relevant to the conceptual aims of the article rather than conducting exhaustive evidence retrieval and may therefore be subject to selection bias.

## 11. Conclusions

The evidence reviewed in this article shows that AMR is shaped by a broad and interconnected set of determinants that operate across One Health systems and often converge through common pathways influencing infection burden, antimicrobial exposure, selection, transmission, and the capacity to prevent and control resistance. The central challenge is therefore not simply to identify individual determinants, but to understand how they interact and where meaningful intervention is possible. This broader perspective moves AMR beyond an antibiotic-centered interpretation and places greater emphasis on prevention before antimicrobial use becomes necessary. Contemporary AMR literacy should accordingly extend towards a One Health perspective that includes an understanding of these interconnections, the limits of individual action, and the different capacities of actors to influence AMR. Knowledge alone cannot overcome structural barriers when the conditions required for appropriate action are absent. Shared responsibility should therefore be understood as differentiated responsibility among individuals, health professionals, institutions, industry, and governments. Preserving effective antimicrobials will depend on sustained and coordinated action across One Health sectors, with prevention, stewardship, surveillance, and system strengthening treated as connected components of the same response.

## Figures and Tables

**Table 1:** Selected AMR determinants, main pathways, critical points for intervention, and preventive actions across One Health contexts.

Context	AMR Determinant	Main Pathway to AMR	Critical Point for Intervention	Potential Intervention	Expected Contribution to AMR Prevention and Control	Selected References
Community	Gaps in community infection prevention, including inadequate water, sanitation and hygiene	Increased infection burden and subsequent antimicrobial demand	Before infection occurs or antimicrobial treatment is required	Vaccination, safe water, sanitation and hygiene, community infection prevention	Prevents infections and reduces the need for antimicrobial treatment	[34,35]
Primary care	Diagnostic uncertainty and inappropriate prescribing	Unnecessary or broad-spectrum antimicrobial exposure	At the diagnostic and prescribing decision	Timely microbiological diagnostics, prescribing guidance, antimicrobial stewardship	Supports targeted treatment and reduces unnecessary broad-spectrum antimicrobial exposure	[29]
Secondary and tertiary care	Gaps in infection prevention and control in health care settings	Health care-associated transmission of resistant microorganisms	During patient care and infection prevention procedures	Infection prevention and control programs, hand hygiene, screening and isolation where indicated	Reduces health care-associated infections and transmission of resistant organisms	[6,33]
Livestock	High animal disease burden and antimicrobial use	Increased antimicrobial exposure and selection pressure in animal populations	Before antimicrobial treatment becomes necessary and at veterinary prescribing	Vaccination, biosecurity, hygiene, improved husbandry, diagnostics and responsible antimicrobial use	Reduces animal infections, antimicrobial demand and selection pressure	[45,50]
Agriculture	Land application of manure or sludge containing antimicrobial residues, resistant microorganisms or resistance genes	Environmental contamination and dissemination through soil and water	Before land application and subsequent environmental dispersal	Treatment and controlled application of manure and sludge, combined with measures to reduce agricultural runoff	Limits environmental dissemination of antimicrobial residues, resistant microorganisms and resistance genes into soil and water	[45,75]
Wastewater	Discharge of antimicrobial residues, resistant microorganisms and resistance genes in human and health care wastewater	Environmental release, persistence and downstream dissemination	Before release of treated effluent into the environment	Improved wastewater treatment and appropriate treatment of high-risk waste streams	Reduces environmental release and downstream dissemination	[54,57]
Pharmaceutical production	Release of antibiotics and other antimicrobial residues in manufacturing effluents	Environmental antimicrobial exposure and selection pressure	At the source and before effluent discharge	Manufacturing emission standards, effluent monitoring and appropriate wastewater treatment	Reduces environmental antimicrobial concentrations and associated selection pressure	[54,75]
Environmental and climate change	Rising temperatures, extreme weather events and climate-related disruption	Increased infection and transmission risk, disruption of water, sanitation and health systems, and increased antimicrobial demand	Response planning	Climate-resilient health and sanitation infrastructure and climate-informed surveillance	Reduces climate-related infection and transmission risks and helps maintain prevention and response capacity during climate-related disruption	[59,60]
Health emergencies	Disruption of diagnostics, surveillance, infection prevention, health services and antimicrobial supply	Reduced stewardship and infection control capacity, with increased risk of inappropriate use and transmission	Emergency preparedness and response planning	Emergency antimicrobial stewardship protocols, continuity of diagnostics, surveillance, infection prevention and essential antimicrobial access	Maintains stewardship, surveillance and infection control capacity during crises	[12,42]
Social and institutional systems	Structural disadvantages, barriers to health care and weak system capacity	Increased infection burden, delayed appropriate care and inappropriate antimicrobial use	Community, health system and policy level	Improved access to health care and sanitation, stronger public health capacity and context-specific prevention	Reduces conditions associated with infection burden, delayed appropriate care and inappropriate antimicrobial use	[76]

## Data Availability

No new data were created or analyzed in this study. Data sharing is not applicable to this article.

## Abbreviations

AMR	Antimicrobial resistance
COVID-19	Coronavirus disease 2019
ECDC	European Centre for Disease Prevention and Control
FAO	Food and Agriculture Organization of the United Nations
UNEP	United Nations Environment Programme
WOAH	World Organization for Animal Health
WHO	World Health Organization
